# Supplementary material for: Evolution of genes involved in feeding preference and metabolic processes in Calliphoridae (Diptera: Calyptratae)
Source: PeerJ. 2016 Oct 27;4:e2598. doi: 10.7717/peerj.2598 (PMC5088637; doi:10.7717/peerj.2598)
Supplement: Table S1 [file peerj-04-2598-s001.pdf]

Table S1. Candidate genes potentially involved in feeding habit.

Gene	Symbol	Accession[1]	GO ID	GO term	Reference
Cyp6g1	*Cyp6g1*	KF432201	0017085	"Response to insecticide"	[1] Joussen et al. 2008
foraging	*for*	KF432198	0030536	"Larval feeding behavior"	[2] Fitzpatrick & Sokolowski 2004
Glutamate dehydrogenase	*Gdh*	KF432200	0006536	"Glutamate metabolic process"	[3] Bond and Sang 1968
Jonah65Aiv	*Jon65Aiv*	KF432196	0004252	"Serine-type endopeptidase activity"	[4] Ross et al. 2003
Malvolio	*Mvl*	KF432194	0050916	"Sensory perception of sweet taste"	[5] Orgad et al. 1998
PGRP-SC	*PGRP-SC2*	KF432195	0006955	"Immune response"	[6] Werner et al. 2000
RPS6-p70-protein kinase	*S6k*	KF432199	0030536	"Larval feeding behavior"	[7] Wu et al. 2005
Smooth	*sm*	KF432197	008343	"Adult feeding behavior"	[8] Layalle et al. 2005

[1] Genbank accession numbers for *Co. hominivorax* sequence used for primer design

[1] Joussen N, Heckel DG, Haas M, Schuphan I, and Schmidt B. 2008. Metabolism of imidacloprid and DDT by P450 CYP6G1 expressed in cell cultures of Nicotiana tabacum suggests detoxification of these insecticides in Cyp6g1-overexpressing strains of Drosophila melanogaster, leading to resistance. *Pest Manag Sci* 64:65-73. 10.1002/ps.1472.

[2] Fitzpatrick MJ, and Sokolowski MB. 2004. In Search of Food: Exploring the Evolutionary Link Between cGMP-Dependent Protein Kinase (PKG) and *Behaviour. Integr Comp Biol* 44:28-36. 10.1093/icb/44.1.28.

[3] Bond PA, and Sang JH. 1968. Glutamate dehydrogenase of Drosophila larvae. *J Insect Physiol* 14:341-359.

[4] Ross J, Jiang H, Kanost MR, and Wang Y. 2003. Serine proteases and their homologs in the Drosophila melanogaster genome: an initial analysis of sequence conservation and phylogenetic relationships. *Gene* 304:117-131.

[5] Orgad S, Nelson H, Segal D, and Nelson N. 1998. Metal ions suppress the abnormal taste behavior of the Drosophila mutant malvolio. *J Exp Biol* 201:115-120.

[6] Werner T, Liu G, Kang D, Ekengren S, Steiner H, and Hultmark D. 2000. A family of peptidoglycan recognition proteins in the fruit fly Drosophila melanogaster. *Proc Natl Acad Sci U S A* 97:13772-13777. 10.1073/pnas.97.25.13772.

[7] Wu Q, Zhang Y, Xu J, and Shen P. 2005. Regulation of hunger-driven behaviors by neural ribosomal S6 kinase in Drosophila. *Proc Natl Acad Sci U S A* 102:13289-13294. 10.1073/pnas.0501914102.

[8] Layalle S, Coessens E, Ghysen A, and Dambly-Chaudiere C. 2005. Smooth, a hnRNP encoding gene, controls axonal navigation in Drosophila. *Genes Cells* 10:119-125. 10.1111/j.1365-2443.2005.00822.x.
