# Supplementary material for: Evolution of genes involved in feeding preference and metabolic processes in Calliphoridae (Diptera: Calyptratae)
Source: PeerJ. 2016 Oct 27;4:e2598. doi: 10.7717/peerj.2598 (PMC5088637; doi:10.7717/peerj.2598)
Supplement: Table S2 [file peerj-04-2598-s002.pdf]

Table S2. Candidate genes that failed to amplify in this study. Several genes were selected for their potential involvement in feeding habits. However, some of these genes were not pursued in our analysis because we were not able to successfully amplify them in all target species.

Gene	Symbol	GO ID	GO term	Category[1]	References
AlphaTrypsin	*Alphatry*	0006508	"Proteolysis"	d	[1] Ross et al. 2003
Attacin-A	*AttA*	0019731	"Antibacterial humoral response "	c	[2] Lemaitre et al. 1997
calcium-binding protein 1	*Cabp1*	0003756	"Protein disulfide isomerase activity"	b	[3] Ashburn et al. 1999
Cyp12a4	*Cyp12a4*	0055114	"Oxidation-reduction process"	b	[4] Bogwitz et al. 2005
Cyp4d14	*Cyp4d14*	0055114	"Oxidation-reduction process"	b	[5] FlyBase et al. 2004
Diptericin A	*DptA*	0009617	" Response to bacterium"	c	[6] Berkey et al. 2009
Hemolectin	*Hml*	0042060	"Wound healing"	c	[7] Lesch et al. 2007
neuropeptide F	*NPF*	0030536	"Larval feeding behavior"	a	[8] Wu et al. 2003
ovo	*ovo*	0008343	"Adult feeding behavior"	a	[9] Wong et al. 2009
painless	*pain*	0042048	"Olfactory behavior"	a	[10] Wang et al. 2011
scalloped	*sd*	0007423	"Sensory organ development"	a	[11] Srivastava & Bell. 2003
Serpin 55B	*Spn55B*	0045861	"Negative regulation of proteolysis"	d	[12] Han et al. 2000
shibire	*shi*	0030536	"Larval feeding behavior"	a	[13] Wu et al. 2005

[1] The selected genes can be classified in four categories: (a) genes involved in feeding preference; (b) genes involved in toxin metabolism that act in food detoxification; (c) genes involved in immune responses; and (d) genes involved in wound formation that allow larvae to move around the wound during feeding.

[1] Ross J, Jiang H, Kanost MR, and Wang Y. 2003. Serine proteases and their homologs in the Drosophila melanogaster genome: an initial analysis of sequence conservation and phylogenetic relationships. *Gene* 304:117-131.

[2] Lemaitre B, Reichhart JM, and Hoffmann JA. 1997. Drosophila host defense: differential induction of antimicrobial peptide genes after infection by various classes of microorganisms. *Proc Natl Acad Sci U S A* 94:14614-14619.

[3] Ashburner M, Misra S, Roote J, Lewis SE, Blazej R, Davis T, Doyle C, Galle R, George R, Harris N, Hartzell G, Harvey D, Hong L, Houston K, Hoskins R, Johnson G, Martin C, Moshrefi A, Palazzolo M, Reese MG, Spradling A, Tsang G, Wan K, Whitelaw K, Celniker S, and et al. 1999. An exploration of the sequence of a 2.9-Mb region of the genome of Drosophila melanogaster: the Adh region. *Genetics* 153:179-219.

[4] Bogwitz MR, Chung H, Magoc L, Rigby S, Wong W, O'Keefe M, McKenzie JA, Batterham P, and Daborn PJ. 2005. Cyp12a4 confers lufenuron resistance in a natural population of Drosophila melanogaster. *Proc Natl Acad Sci U S A* 102:12807-12812. 10.1073/pnas.0503709102

[5] FlyBase C, Swiss-Prot Project M, and InterPro Project M. 2004. Gene Ontology annotation in FlyBase through association of InterPro records with GO terms.

[6] Berkey CD, Blow N, and Watnick PI. 2009. Genetic analysis of Drosophila melanogaster susceptibility to intestinal Vibrio cholerae infection. *Cell Microbiol* 11:461-474. 10.1111/j.1462-5822.2008.01267.x

[7] Lesch C, Goto A, Lindgren M, Bidla G, Dushay MS, and Theopold U. 2007. A role for Hemolectin in coagulation and immunity in Drosophila melanogaster. *Dev Comp Immunol* 31:1255-1263.

[8] Wu Q, Wen T, Lee G, Park JH, Cai HN, and Shen P. 2003. Developmental control of foraging and social behavior by the Drosophila neuropeptide Y-like system. *Neuron* 39:147-161.

[9] Wong R, Piper MD, Wertheim B, and Partridge L. 2009. Quantification of food intake in Drosophila. *PLoS One* 4:e6063. 10.1371/journal.pone.0006063

[10] Wang K, Guo Y, Wang F, and Wang Z. 2011. Drosophila TRPA channel painless inhibits male-male courtship behavior through modulating olfactory sensation. *PLoS One* 6:e25890. 10.1371/journal.pone.0025890

[11] Srivastava A, and Bell JB. 2003. Further developmental roles of the Vestigial/Scalloped transcription complex during wing development in Drosophila melanogaster. *Mech Dev* 120:587-596.

[12] Han J, Zhang H, Min G, Kemler D, and Hashimoto C. 2000. A novel Drosophila serpin that inhibits serine proteases. *FEBS Lett* 468:194-198.

[13] Wu Q, Zhang Y, Xu J, and Shen P. 2005. Regulation of hunger-driven behaviors by neural ribosomal S6 kinase in Drosophila. *Proc Natl Acad Sci U S A* 102:13289-13294. 10.1073/pnas.0501914102.
