# Supplementary material for: Evolution of genes involved in feeding preference and metabolic processes in Calliphoridae (Diptera: Calyptratae)
Source: PeerJ. 2016 Oct 27;4:e2598. doi: 10.7717/peerj.2598 (PMC5088637; doi:10.7717/peerj.2598)
Supplement: Table S3 [file peerj-04-2598-s003.pdf]

Table S3. qPCR primer efficiency.

Gene	[1] Primer sequence (5' --> 3')	Species	Efficiency
Cyp6g1	F: GAGCCCGATAAATTCGAACC<br>R: ATCATGCCAAAACGTTCACC	*Ch. albiceps*	104%
		Ch. megacephala	98%
		Co. hominivorax	97%
		Co. macellaria	98%
foraging	F: TGGTGGGGTTTACAAAATCG<br>R: TTATCCCAGCCGGTAACATC	*Ch. albiceps*	96%
		Ch. megacephala	100%
		Co. hominivorax	100%
		Co. macellaria	106%
Glutamate dehydrogenase	F: CATTCGGTGGTGCTAAGG<br>R: CCGGGTCCAATGAAACC	*Ch. albiceps*	105%
		Ch. megacephala	96%
		Co. hominivorax	107%
		Co. macellaria	95%
Jonah65Aiv	F: ACTGCTGCTCATTGTACTGATG<br>R: TTGCTATTCCAGCCAGAGTG	*Ch. albiceps*	98%
		Ch. megacephala	95%
		Co. hominivorax	100%
		Co. macellaria	99%
Malvolio	F: TGGGGTGTGGGTATTTTAGC<br>R: TAAAACACGACGCCAACG	*Ch. albiceps*	91%
		Ch. megacephala	91%[2]
		Co. hominivorax	97%
		Co. macellaria	96%
PGRP-SC2	F: CGTGGCTGGAATGTTATGG<br>R: TTGACCACGAGCAACAGC	*Ch. albiceps*	98%
		Ch. megacephala	91%
		Co. hominivorax	97%
		Co. macellaria	98%
RPS6-p70-protein kinase	F: TCACCAAGACGTACACCAAG<br>R: TGTACATCCATCATTTCGTC	*Ch. albiceps*	98%
		Ch. megacephala	100%
		Co. hominivorax	98%
		Co. macellaria	98%
Smooth	F: TTCTTATTGCCCGATCATACAC<br>R: AATCTTGCTCGGTGGTTG	*Ch. albiceps*	98%
		Ch. megacephala	99%
		Co. hominivorax	96%
		Co. macellaria	95%

[1] Primer sequences (F= forward; R= reverse)

[2] *Ch.albiceps* efficiency was used to calculate mRNA levels of *Ch. megacephala*.
