# Supplementary material for: Evolution of genes involved in feeding preference and metabolic processes in Calliphoridae (Diptera: Calyptratae)
Source: PeerJ. 2016 Oct 27;4:e2598. doi: 10.7717/peerj.2598 (PMC5088637; doi:10.7717/peerj.2598)
Supplement: Table S4 [file peerj-04-2598-s004.pdf]

Table S4. Sequencing primers.

Gene	Primer (5' -> 3')[1]	Tm (ºC)	Length (bp)
foraging	F1: TCCCAAATACGATAAGGATG	55.11	821
	R1: CATCACGGAATTCTTCATTG	56.55	
	F2: ACTTGCTTGGTCATAGATCG	55.41	946
	R2: TTTGTAAACCCCACCAATAG	55.12	
Glutamate dehydrogenase	F1: AAAGGCTTTGCCTACTGCTG	59.65	975
	R1: TGGTACCGTGTTCGTTCTTG	59.61	
	F2: AACGAAGCCAACTACATGAGC	59.40	808
	R2: CATCACGGTAAGTGGTGAAG	56.59	
Jonah65Aiv	F: GCCTGGTGTGGTGGTTC	57.00	621
	R: CGATACCAGTATTAGATTTAATCC	57.00	
Malvolio	F1: CGTAAATTATGGGCCTTCAC	56.59	836
	R1: GAATGTTTGGCATCTTCRTAC	57.14	
	F2: GTTTTTGCTCATGGCATG	56.03	661
	R2: CGGCCATATGTATCACAAG	54.93	
RPS6-p70-protein kinase	F1: ATGGCTGACGTGASYG	51.18	790
	R1: AAGCACCTAATGACCACC	53.23	
	F2: CGTGGTCATGGTAAAGC	53.01	644
	R2: TGTACATCCATCATTTCGTC	54.21	

[1] Primer sequences (F= forward; R= reverse). For sequences amplified in two discontinuous segments: F1= first forward fragment; R1= first reverse fragment; F2= second forward fragment; R2= second reverse fragment;)
