# Supplementary material for: Evolution of genes involved in feeding preference and metabolic processes in Calliphoridae (Diptera: Calyptratae)
Source: PeerJ. 2016 Oct 27;4:e2598. doi: 10.7717/peerj.2598 (PMC5088637; doi:10.7717/peerj.2598)
Supplement: Table S5 [file peerj-04-2598-s005.pdf]

Table S5. NCBI accession numbers of the sequences generated.

Gene	Species	NCBI Accession
Jon65aiv	*Ch. megacephala*	KU234347
	Ch. megacephala	KU234348
	Ch. albiceps	KU234349
	Ch. albiceps	KU234350
	Co. macellaria	KU234351
	Co. macellaria	KU234352
	Co. hominivorax	KU234353
	Co. hominivorax	KU234354
for	*Co. hominivorax*	KU234355
	Co. hominivorax	KU234356
	Co. macellaria	KU234357
	Co. macellaria	KU234358
	Ch. albiceps	KU234359
	Ch. albiceps	KU234360
	Ch. megacephala	KU234361
	Ch. megacephala	KU234362
Gdh	*Co. macellaria*	KU234363
	Co. macellaria	KU234364
	Ch. megacephala	KU234365
	Ch. megacephala	KU234366
	Ch. albiceps	KU234367
	Ch. albiceps	KU234368
	Co. hominivorax	KU234369
	Co. hominivorax	KU234370
S6k	*Ch. megacephala*	KU234371
	Co. macellaria	KU234372
	Co. macellaria	KU234373
	Co. hominivorax	KU234374
	Co. hominivorax	KU234375
	Ch. albiceps	KU234376
	Ch. albiceps	KU234377
	Ch. megacephala	KU234378
Mvl	*D. hominis*	KU234379
	Ch. megacephala	KU234380
	Ch. megacephala	KU234381
	Co. macellaria	KU234382
	Co. macellaria	KU234383
	Co. hominivorax	KU234384
	Co. hominivorax	KU234385
	Ch. albiceps	KU234386
	O. ovis	KU234387
	Ch. albiceps	KU234388
