# Supplementary material for: Evolution of genes involved in feeding preference and metabolic processes in Calliphoridae (Diptera: Calyptratae)
Source: PeerJ. 2016 Oct 27;4:e2598. doi: 10.7717/peerj.2598 (PMC5088637; doi:10.7717/peerj.2598)
Supplement: Table S6 [file peerj-04-2598-s006.pdf]

Table S6. Gene expression levels of the candidate genes as measured by qPCR.

Species	Stage/Sex	Gene	Replicate	MNE (*Rp49*)	MNE (*Gapdh*)	Average MNE
Ch. albiceps	Adult females	*Cyp6g1*	1	0.00081	0.00052	0.00066
Ch. albiceps	Adult females	*Cyp6g1*	2	0.00071	0.00049	0.00060
Ch. albiceps	Adult females	*Cyp6g1*	3	0.00073	0.00019	0.00046
Ch. albiceps	Adult females	*Cyp6g1*	4	0.00157	0.00038	0.00098
Co. hominivorax	Adult females	*Cyp6g1*	1	0.02848	0.02551	0.02700
Co. hominivorax	Adult females	*Cyp6g1*	2	0.03770	0.01896	0.02833
Co. hominivorax	Adult females	*Cyp6g1*	3	0.00411	0.00195	0.00303
Co. hominivorax	Adult females	*Cyp6g1*	4	0.00397	0.00245	0.00321
Co. macellaria	Adult females	*Cyp6g1*	1	0.02507	0.01361	0.01934
Co. macellaria	Adult females	*Cyp6g1*	2	0.00660	0.00339	0.00499
Co. macellaria	Adult females	*Cyp6g1*	3	0.00636	0.00224	0.00430
Co. macellaria	Adult females	*Cyp6g1*	4	0.00358	0.00134	0.00246
Ch. megacephala	Adult females	*Cyp6g1*	1	0.00539	0.00222	0.00381
Ch. megacephala	Adult females	*Cyp6g1*	2	0.00705	0.00352	0.00528
Ch. megacephala	Adult females	*Cyp6g1*	3	0.00598	0.00222	0.00410
Ch. megacephala	Adult females	*Cyp6g1*	4	0.00300	0.00105	0.00203
Ch. albiceps	Larvae	*Cyp6g1*	1	0.01727	0.00418	0.01072
Ch. albiceps	Larvae	*Cyp6g1*	2	0.08465	0.01880	0.05172
Ch. albiceps	Larvae	*Cyp6g1*	3	0.08452	0.03585	0.06018
Ch. albiceps	Larvae	*Cyp6g1*	4	0.07243	0.03627	0.05435
Co. hominivorax	Larvae	*Cyp6g1*	1	0.15789	0.35147	0.25468
Co. hominivorax	Larvae	*Cyp6g1*	2	0.16684	0.25539	0.21112
Co. hominivorax	Larvae	*Cyp6g1*	3	0.00723	0.00293	0.00508
Co. hominivorax	Larvae	*Cyp6g1*	4	0.00644	0.00274	0.00459
Co. macellaria	Larvae	*Cyp6g1*	1	0.00278	0.00323	0.00300
Co. macellaria	Larvae	*Cyp6g1*	2	0.00349	0.00524	0.00436
Co. macellaria	Larvae	*Cyp6g1*	3	0.01264	0.00406	0.00835
Co. macellaria	Larvae	*Cyp6g1*	4	0.02960	0.00895	0.01927
Ch. megacephala	Larvae	*Cyp6g1*	1	0.02166	0.00437	0.01301
Ch. megacephala	Larvae	*Cyp6g1*	2	0.09460	0.03125	0.06293
Ch. megacephala	Larvae	*Cyp6g1*	3	0.09401	0.03451	0.06426
Ch. megacephala	Larvae	*Cyp6g1*	4	0.07787	0.02189	0.04988
Ch. albiceps	Adult males	*Cyp6g1*	1	0.00412	0.00159	0.00285
Ch. albiceps	Adult males	*Cyp6g1*	2	0.00046	0.00020	0.00033
Ch. albiceps	Adult males	*Cyp6g1*	3	0.00488	0.00110	0.00299
Ch. albiceps	Adult males	*Cyp6g1*	4	0.01686	0.00340	0.01013
Co. hominivorax	Adult males	*Cyp6g1*	1	0.04040	0.01408	0.02724
Co. hominivorax	Adult males	*Cyp6g1*	2	0.02486	0.01116	0.01801
Co. hominivorax	Adult males	*Cyp6g1*	3	0.00390	0.00148	0.00269
Co. hominivorax	Adult males	*Cyp6g1*	4	0.00474	0.00299	0.00387
Co. macellaria	Adult males	*Cyp6g1*	1	0.01013	0.00423	0.00718
Co. macellaria	Adult males	*Cyp6g1*	2	0.01006	0.00370	0.00688
Co. macellaria	Adult males	*Cyp6g1*	3	0.00769	0.00232	0.00501
Co. macellaria	Adult males	*Cyp6g1*	4	0.00877	0.00305	0.00591
Ch. megacephala	Adult males	*Cyp6g1*	1	0.00852	0.00262	0.00557
Ch. megacephala	Adult males	*Cyp6g1*	2	0.00911	0.00286	0.00598
Ch. megacephala	Adult males	*Cyp6g1*	3	0.00791	0.00262	0.00527
Ch. megacephala	Adult males	*Cyp6g1*	4	0.00494	0.00178	0.00336
Ch. albiceps	Adult females	*for*	1	0.10285	0.06726	0.08505
Ch. albiceps	Adult females	*for*	2	0.09130	0.05558	0.07344

Ch. albiceps	Adult females	*for*	3	0.13475	0.03548	0.08512
Ch. albiceps	Adult females	*for*	4	0.08726	0.02348	0.05537
Co. hominivorax	Adult females	*for*	1	0.08221	0.06083	0.07152
Co. hominivorax	Adult females	*for*	2	0.11185	0.05839	0.08512
Co. hominivorax	Adult females	*for*	3	0.05810	0.02803	0.04307
Co. hominivorax	Adult females	*for*	4	0.05980	0.02851	0.04416
Co. macellaria	Adult females	*for*	1	0.08256	0.04790	0.06523
Co. macellaria	Adult females	*for*	2	0.07519	0.04634	0.06076
Co. macellaria	Adult females	*for*	3	0.06725	0.02051	0.04388
Co. macellaria	Adult females	*for*	4	0.07420	0.02562	0.04991
Ch. megacephala	Adult females	*for*	1	0.02723	0.01418	0.02070
Ch. megacephala	Adult females	*for*	2	0.02255	0.01660	0.01958
Ch. megacephala	Adult females	*for*	3	0.02553	0.00794	0.01673
Ch. megacephala	Adult females	*for*	4	0.03430	0.01091	0.02260
Ch. albiceps	Larvae	*for*	1	0.02445	0.00654	0.01549
Ch. albiceps	Larvae	*for*	2	0.01788	0.00454	0.01121
Ch. albiceps	Larvae	*for*	3	0.01287	0.00462	0.00874
Ch. albiceps	Larvae	*for*	4	0.01369	0.00554	0.00962
Co. hominivorax	Larvae	*for*	1	0.02353	0.04089	0.03221
Co. hominivorax	Larvae	*for*	2	0.02436	0.04094	0.03265
Co. hominivorax	Larvae	*for*	3	0.01409	0.00625	0.01017
Co. hominivorax	Larvae	*for*	4	0.01970	0.00675	0.01323
Co. macellaria	Larvae	*for*	1	0.02717	0.03928	0.03323
Co. macellaria	Larvae	*for*	2	0.02283	0.04779	0.03531
Co. macellaria	Larvae	*for*	3	0.01581	0.00478	0.01029
Co. macellaria	Larvae	*for*	4	0.01583	0.00460	0.01021
Ch. megacephala	Larvae	*for*	1	0.00604	0.00155	0.00379
Ch. megacephala	Larvae	*for*	2	0.00401	0.00191	0.00296
Ch. megacephala	Larvae	*for*	3	0.00404	0.00108	0.00256
Ch. megacephala	Larvae	*for*	4	0.00787	0.00174	0.00480
Ch. albiceps	Adult males	*for*	1	0.13815	0.04509	0.09162
Ch. albiceps	Adult males	*for*	2	0.11454	0.04798	0.08126
Ch. albiceps	Adult males	*for*	3	0.05156	0.01144	0.03150
Ch. albiceps	Adult males	*for*	4	0.06495	0.01300	0.03898
Co. hominivorax	Adult males	*for*	1	0.09069	0.03022	0.06046
Co. hominivorax	Adult males	*for*	2	0.10033	0.04076	0.07054
Co. hominivorax	Adult males	*for*	3	0.06223	0.02025	0.04124
Co. hominivorax	Adult males	*for*	4	0.06609	0.03595	0.05102
Co. macellaria	Adult males	*for*	1	0.07825	0.03298	0.05562
Co. macellaria	Adult males	*for*	2	0.06516	0.03150	0.04833
Co. macellaria	Adult males	*for*	3	0.08946	0.02184	0.05565
Co. macellaria	Adult males	*for*	4	0.07478	0.02717	0.05097
Ch. megacephala	Adult males	*for*	1	0.04085	0.01439	0.02762
Ch. megacephala	Adult males	*for*	2	0.03775	0.01699	0.02737
Ch. megacephala	Adult males	*for*	3	0.03413	0.01101	0.02257
Ch. megacephala	Adult males	*for*	4	0.04069	0.01173	0.02621
Ch. albiceps	Adult females	*Gdh*	1	0.00630	0.00391	0.00510
Ch. albiceps	Adult females	*Gdh*	2	0.00432	0.00217	0.00324
Ch. albiceps	Adult females	*Gdh*	3	0.04451	0.01172	0.02811
Ch. albiceps	Adult females	*Gdh*	4	0.26317	0.09019	0.17668
Co. hominivorax	Adult females	*Gdh*	1	0.00087	0.00064	0.00075
Co. hominivorax	Adult females	*Gdh*	2	0.00757	0.00429	0.00593

Co. hominivorax	Adult females	*Gdh*	3	0.02468	0.01191	0.01829
Co. hominivorax	Adult females	*Gdh*	4	0.30920	0.08982	0.19951
Co. macellaria	Adult females	*Gdh*	1	0.00656	0.00381	0.00519
Co. macellaria	Adult females	*Gdh*	2	0.01083	0.00659	0.00871
Co. macellaria	Adult females	*Gdh*	3	0.09832	0.02998	0.06415
Co. macellaria	Adult females	*Gdh*	4	0.09264	0.03751	0.06508
Ch. megacephala	Adult females	*Gdh*	1	0.02531	0.01318	0.01924
Ch. megacephala	Adult females	*Gdh*	2	0.05681	0.04181	0.04931
Ch. megacephala	Adult females	*Gdh*	3	0.06078	0.01889	0.03984
Ch. megacephala	Adult females	*Gdh*	4	0.29164	0.06440	0.17802
Ch. albiceps	Larvae	*Gdh*	1	0.14810	0.03958	0.09384
Ch. albiceps	Larvae	*Gdh*	2	0.03119	0.05241	0.04180
Ch. albiceps	Larvae	*Gdh*	3	0.09174	0.03295	0.06234
Ch. albiceps	Larvae	*Gdh*	4	0.02818	0.01344	0.02081
Co. hominivorax	Larvae	*Gdh*	1	0.04346	0.07553	0.05949
Co. hominivorax	Larvae	*Gdh*	2	0.03604	0.07545	0.05574
Co. hominivorax	Larvae	*Gdh*	3	0.24137	0.10708	0.17423
Co. hominivorax	Larvae	*Gdh*	4	0.10675	0.03686	0.07181
Co. macellaria	Larvae	*Gdh*	1	0.10407	0.15046	0.12727
Co. macellaria	Larvae	*Gdh*	2	0.15613	0.03968	0.09790
Co. macellaria	Larvae	*Gdh*	3	0.28664	0.08658	0.18661
Co. macellaria	Larvae	*Gdh*	4	0.04153	0.01118	0.02635
Ch. megacephala	Larvae	*Gdh*	1	0.22734	0.05823	0.14279
Ch. megacephala	Larvae	*Gdh*	2	0.05391	0.02575	0.03983
Ch. megacephala	Larvae	*Gdh*	3	0.17371	0.04623	0.10997
Ch. megacephala	Larvae	*Gdh*	4	0.06425	0.02044	0.04234
Ch. albiceps	Adult males	*Gdh*	1	0.00544	0.00187	0.00366
Ch. albiceps	Adult males	*Gdh*	2	0.00759	0.00309	0.00534
Ch. albiceps	Adult males	*Gdh*	3	0.03224	0.00715	0.01970
Ch. albiceps	Adult males	*Gdh*	4	0.05526	0.01106	0.03316
Co. hominivorax	Adult males	*Gdh*	1	0.01554	0.00518	0.01036
Co. hominivorax	Adult males	*Gdh*	2	0.02177	0.01053	0.01615
Co. hominivorax	Adult males	*Gdh*	3	0.02340	0.00761	0.01550
Co. hominivorax	Adult males	*Gdh*	4	0.00645	0.00351	0.00498
Co. macellaria	Adult males	*Gdh*	1	0.07724	0.03255	0.05490
Co. macellaria	Adult males	*Gdh*	2	0.01336	0.00554	0.00945
Co. macellaria	Adult males	*Gdh*	3	0.08595	0.02099	0.05347
Co. macellaria	Adult males	*Gdh*	4	0.04161	0.01512	0.02836
Ch. megacephala	Adult males	*Gdh*	1	0.05155	0.01816	0.03485
Ch. megacephala	Adult males	*Gdh*	2	0.01861	0.00838	0.01349
Ch. megacephala	Adult males	*Gdh*	3	0.04811	0.01552	0.03181
Ch. megacephala	Adult males	*Gdh*	4	0.05938	0.01712	0.03825
Ch. albiceps	Adult females	*Jon65aiv*	1	0.00227	0.00146	0.00187
Ch. albiceps	Adult females	*Jon65aiv*	2	0.00683	0.00466	0.00575
Ch. albiceps	Adult females	*Jon65aiv*	3	0.00689	0.00182	0.00436
Ch. albiceps	Adult females	*Jon65aiv*	4	0.00507	0.00123	0.00315
Co. hominivorax	Adult females	*Jon65aiv*	1	0.15156	0.11087	0.13122
Co. hominivorax	Adult females	*Jon65aiv*	2	0.53839	0.26522	0.40180
Co. hominivorax	Adult females	*Jon65aiv*	3	0.37937	0.17987	0.27962
Co. hominivorax	Adult females	*Jon65aiv*	4	0.42129	0.25970	0.34050
Co. macellaria	Adult females	*Jon65aiv*	1	0.07427	0.03354	0.05391
Co. macellaria	Adult females	*Jon65aiv*	2	0.12674	0.06502	0.09588

Co. macellaria	Adult females	*Jon65aiv*	3	0.43095	0.15204	0.29149
Co. macellaria	Adult females	*Jon65aiv*	4	0.41125	0.15332	0.28228
Ch. megacephala	Adult females	*Jon65aiv*	1	0.42242	0.17417	0.29829
Ch. megacephala	Adult females	*Jon65aiv*	2	0.10223	0.04499	0.07361
Ch. megacephala	Adult females	*Jon65aiv*	3	0.15083	0.05608	0.10345
Ch. megacephala	Adult females	*Jon65aiv*	4	0.13207	0.03546	0.08376
Ch. albiceps	Larvae	*Jon65aiv*	1	0.85763	0.20767	0.53265
Ch. albiceps	Larvae	*Jon65aiv*	2	0.72934	0.16197	0.44566
Ch. albiceps	Larvae	*Jon65aiv*	3	0.30904	0.13107	0.22005
Ch. albiceps	Larvae	*Jon65aiv*	4	0.32446	0.16250	0.24348
Co. hominivorax	Larvae	*Jon65aiv*	1	0.96160	2.14059	1.55110
Co. hominivorax	Larvae	*Jon65aiv*	2	1.14360	1.75063	1.44711
Co. hominivorax	Larvae	*Jon65aiv*	3	0.04449	0.01801	0.03125
Co. hominivorax	Larvae	*Jon65aiv*	4	0.06325	0.02693	0.04509
Co. macellaria	Larvae	*Jon65aiv*	1	1.06994	1.41538	1.24266
Co. macellaria	Larvae	*Jon65aiv*	2	0.00364	0.00546	0.00455
Co. macellaria	Larvae	*Jon65aiv*	3	0.12676	0.04071	0.08374
Co. macellaria	Larvae	*Jon65aiv*	4	0.06154	0.01861	0.04007
Ch. megacephala	Larvae	*Jon65aiv*	1	1.24367	0.25086	0.74726
Ch. megacephala	Larvae	*Jon65aiv*	2	0.81681	0.26985	0.54333
Ch. megacephala	Larvae	*Jon65aiv*	3	1.34242	0.49284	0.91763
Ch. megacephala	Larvae	*Jon65aiv*	4	1.31335	0.36915	0.84125
Ch. albiceps	Adult males	*Jon65aiv*	1	0.00396	0.00153	0.00275
Ch. albiceps	Adult males	*Jon65aiv*	2	0.01036	0.00452	0.00744
Ch. albiceps	Adult males	*Jon65aiv*	3	0.01746	0.00395	0.01070
Ch. albiceps	Adult males	*Jon65aiv*	4	0.02121	0.00428	0.01274
Co. hominivorax	Adult males	*Jon65aiv*	1	0.30371	0.10966	0.20669
Co. hominivorax	Adult males	*Jon65aiv*	2	0.17871	0.08020	0.12945
Co. hominivorax	Adult males	*Jon65aiv*	3	0.45034	0.17138	0.31086
Co. hominivorax	Adult males	*Jon65aiv*	4	0.24450	0.15395	0.19922
Co. macellaria	Adult males	*Jon65aiv*	1	0.11136	0.04653	0.07895
Co. macellaria	Adult males	*Jon65aiv*	2	0.22366	0.08227	0.15297
Co. macellaria	Adult males	*Jon65aiv*	3	0.53155	0.16015	0.34585
Co. macellaria	Adult males	*Jon65aiv*	4	0.38860	0.13510	0.26185
Ch. megacephala	Adult males	*Jon65aiv*	1	0.18894	0.05803	0.12349
Ch. megacephala	Adult males	*Jon65aiv*	2	0.15258	0.04791	0.10025
Ch. megacephala	Adult males	*Jon65aiv*	3	0.22454	0.07436	0.14945
Ch. megacephala	Adult males	*Jon65aiv*	4	0.13072	0.04700	0.08886
Ch. albiceps	Adult females	*Mvl*	1	0.00007	0.00004	0.00006
Ch. albiceps	Adult females	*Mvl*	2	0.00012	0.00008	0.00010
Ch. albiceps	Adult females	*Mvl*	3	0.00009	0.00002	0.00005
Ch. albiceps	Adult females	*Mvl*	4	0.00016	0.00004	0.00010
Co. hominivorax	Adult females	*Mvl*	1	0.02277	0.02039	0.02158
Co. hominivorax	Adult females	*Mvl*	2	0.02184	0.01098	0.01641
Co. hominivorax	Adult females	*Mvl*	3	0.01539	0.00770	0.01155
Co. hominivorax	Adult females	*Mvl*	4	0.01392	0.00736	0.01064
Co. macellaria	Adult females	*Mvl*	1	0.00396	0.00233	0.00315
Co. macellaria	Adult females	*Mvl*	2	0.00013	0.00009	0.00011
Co. macellaria	Adult females	*Mvl*	3	0.00409	0.00121	0.00265
Co. macellaria	Adult females	*Mvl*	4	0.00404	0.00115	0.00260
Ch. megacephala	Adult females	*Mvl*	1	0.00004	0.00002	0.00003
Ch. megacephala	Adult females	*Mvl*	2	0.01290	0.00671	0.00980

Ch. megacephala	Adult females	*Mvl*	3	0.00010	0.00004	0.00007
Ch. megacephala	Adult females	*Mvl*	4	0.00016	0.00005	0.00011
Ch. albiceps	Larvae	*Mvl*	1	0.00005	0.00001	0.00003
Ch. albiceps	Larvae	*Mvl*	2	0.00005	0.00001	0.00003
Ch. albiceps	Larvae	*Mvl*	3	0.00016	0.00005	0.00011
Ch. albiceps	Larvae	*Mvl*	4	0.00006	0.00003	0.00005
Co. hominivorax	Larvae	*Mvl*	1	0.00941	0.02094	0.01517
Co. hominivorax	Larvae	*Mvl*	2	0.01245	0.02222	0.01734
Co. hominivorax	Larvae	*Mvl*	3	0.00973	0.00370	0.00671
Co. hominivorax	Larvae	*Mvl*	4	0.00995	0.00367	0.00681
Co. macellaria	Larvae	*Mvl*	1	0.00084	0.00112	0.00098
Co. macellaria	Larvae	*Mvl*	2	0.00072	0.00153	0.00112
Co. macellaria	Larvae	*Mvl*	3	0.00146	0.00041	0.00093
Co. macellaria	Larvae	*Mvl*	4	0.00124	0.00040	0.00082
Ch. megacephala	Larvae	*Mvl*	1	0.00001	0.00000	0.00001
Ch. megacephala	Larvae	*Mvl*	2	0.00004	0.00001	0.00002
Ch. megacephala	Larvae	*Mvl*	3	0.00013	0.00004	0.00008
Ch. megacephala	Larvae	*Mvl*	4	0.00025	0.00006	0.00016
Ch. albiceps	Adult males	*Mvl*	1	0.00064	0.00022	0.00043
Ch. albiceps	Adult males	*Mvl*	2	0.00035	0.00016	0.00025
Ch. albiceps	Adult males	*Mvl*	3	0.00019	0.00004	0.00012
Ch. albiceps	Adult males	*Mvl*	4	0.00033	0.00006	0.00020
Co. hominivorax	Adult males	*Mvl*	1	0.02908	0.01013	0.01960
Co. hominivorax	Adult males	*Mvl*	2	0.00190	0.00075	0.00132
Co. hominivorax	Adult males	*Mvl*	3	0.02282	0.00642	0.01462
Co. hominivorax	Adult males	*Mvl*	4	0.01419	0.00725	0.01072
Co. macellaria	Adult males	*Mvl*	1	0.00375	0.00134	0.00255
Co. macellaria	Adult males	*Mvl*	2	0.00524	0.00168	0.00346
Co. macellaria	Adult males	*Mvl*	3	0.00526	0.00131	0.00329
Co. macellaria	Adult males	*Mvl*	4	0.00443	0.00120	0.00281
Ch. megacephala	Adult males	*Mvl*	1	0.00004	0.00001	0.00003
Ch. megacephala	Adult males	*Mvl*	2	0.00322	0.00121	0.00222
Ch. megacephala	Adult males	*Mvl*	3	0.00072	0.00021	0.00047
Ch. megacephala	Adult males	*Mvl*	4	0.00029	0.00008	0.00019
Ch. albiceps	Adult females	*PGRP-SC2*	1	0.00042	0.00026	0.00034
Ch. albiceps	Adult females	*PGRP-SC2*	2	0.00069	0.00046	0.00058
Ch. albiceps	Adult females	*PGRP-SC2*	3	0.00092	0.00022	0.00057
Ch. albiceps	Adult females	*PGRP-SC2*	4	0.00061	0.00017	0.00039
Co. hominivorax	Adult females	*PGRP-SC2*	1	0.00662	0.00507	0.00584
Co. hominivorax	Adult females	*PGRP-SC2*	2	0.00197	0.00112	0.00154
Co. hominivorax	Adult females	*PGRP-SC2*	3	0.00201	0.00088	0.00145
Co. hominivorax	Adult females	*PGRP-SC2*	4	0.00206	0.00104	0.00155
Co. macellaria	Adult females	*PGRP-SC2*	1	0.01195	0.00649	0.00922
Co. macellaria	Adult females	*PGRP-SC2*	2	0.01261	0.00674	0.00968
Co. macellaria	Adult females	*PGRP-SC2*	3	0.06573	0.01731	0.04152
Co. macellaria	Adult females	*PGRP-SC2*	4	0.06858	0.02099	0.04479
Ch. megacephala	Adult females	*PGRP-SC2*	1	0.21715	0.10816	0.16266
Ch. megacephala	Adult females	*PGRP-SC2*	2	0.09702	0.04505	0.07104
Ch. megacephala	Adult females	*PGRP-SC2*	3	0.15065	0.04214	0.09640
Ch. megacephala	Adult females	*PGRP-SC2*	4	0.05800	0.01842	0.03821
Ch. albiceps	Larvae	*PGRP-SC2*	1	1.11602	0.27657	0.69630
Ch. albiceps	Larvae	*PGRP-SC2*	2	1.51455	0.31801	0.91628

Ch. albiceps	Larvae	*PGRP-SC2*	3	0.46019	0.15597	0.30808
Ch. albiceps	Larvae	*PGRP-SC2*	4	0.77366	0.28627	0.52996
Co. hominivorax	Larvae	*PGRP-SC2*	1	0.57560	1.18369	0.87965
Co. hominivorax	Larvae	*PGRP-SC2*	2	1.20913	2.16664	1.68789
Co. hominivorax	Larvae	*PGRP-SC2*	3	0.50524	0.16338	0.33431
Co. hominivorax	Larvae	*PGRP-SC2*	4	0.86335	0.36547	0.61441
Co. macellaria	Larvae	*PGRP-SC2*	1	2.78721	3.64592	3.21657
Co. macellaria	Larvae	*PGRP-SC2*	2	1.99316	2.73302	2.36309
Co. macellaria	Larvae	*PGRP-SC2*	3	0.63988	0.16789	0.40388
Co. macellaria	Larvae	*PGRP-SC2*	4	0.86422	0.21856	0.54139
Ch. megacephala	Larvae	*PGRP-SC2*	1	3.23650	0.62144	1.92897
Ch. megacephala	Larvae	*PGRP-SC2*	2	2.41886	0.70000	1.55943
Ch. megacephala	Larvae	*PGRP-SC2*	3	4.65001	1.29786	2.97394
Ch. megacephala	Larvae	*PGRP-SC2*	4	3.07321	0.62577	1.84949
Ch. albiceps	Adult males	*PGRP-SC2*	1	0.05885	0.02467	0.04176
Ch. albiceps	Adult males	*PGRP-SC2*	2	0.00205	0.00084	0.00144
Ch. albiceps	Adult males	*PGRP-SC2*	3	0.00583	0.00126	0.00354
Ch. albiceps	Adult males	*PGRP-SC2*	4	0.00257	0.00047	0.00152
Co. hominivorax	Adult males	*PGRP-SC2*	1	0.00346	0.00143	0.00244
Co. hominivorax	Adult males	*PGRP-SC2*	2	0.00161	0.00081	0.00121
Co. hominivorax	Adult males	*PGRP-SC2*	3	0.00270	0.00088	0.00179
Co. hominivorax	Adult males	*PGRP-SC2*	4	0.00901	0.00486	0.00693
Co. macellaria	Adult males	*PGRP-SC2*	1	0.01329	0.00540	0.00935
Co. macellaria	Adult males	*PGRP-SC2*	2	0.00772	0.00333	0.00552
Co. macellaria	Adult males	*PGRP-SC2*	3	0.09918	0.02690	0.06304
Co. macellaria	Adult males	*PGRP-SC2*	4	0.03278	0.00803	0.02041
Ch. megacephala	Adult males	*PGRP-SC2*	1	0.07636	0.02386	0.05011
Ch. megacephala	Adult males	*PGRP-SC2*	2	0.05543	0.01725	0.03634
Ch. megacephala	Adult males	*PGRP-SC2*	3	0.18834	0.04783	0.11808
Ch. megacephala	Adult males	*PGRP-SC2*	4	0.09702	0.02957	0.06329
Ch. albiceps	Adult females	*S6k*	1	0.02389	0.01580	0.01984
Ch. albiceps	Adult females	*S6k*	2	0.03006	0.02036	0.02521
Ch. albiceps	Adult females	*S6k*	3	0.07843	0.02246	0.05044
Ch. albiceps	Adult females	*S6k*	4	0.08574	0.02178	0.05376
Co. hominivorax	Adult females	*S6k*	1	0.09165	0.08208	0.08686
Co. hominivorax	Adult females	*S6k*	2	0.11349	0.05706	0.08528
Co. hominivorax	Adult females	*S6k*	3	0.04556	0.02429	0.03493
Co. hominivorax	Adult females	*S6k*	4	0.05800	0.03286	0.04543
Co. macellaria	Adult females	*S6k*	1	0.06664	0.03800	0.05232
Co. macellaria	Adult females	*S6k*	2	0.06341	0.04008	0.05175
Co. macellaria	Adult females	*S6k*	3	0.05588	0.01551	0.03569
Co. macellaria	Adult females	*S6k*	4	0.07295	0.02031	0.04663
Ch. megacephala	Adult females	*S6k*	1	0.03942	0.02145	0.03043
Ch. megacephala	Adult females	*S6k*	2	0.03972	0.01996	0.02984
Ch. megacephala	Adult females	*S6k*	3	0.04829	0.01589	0.03209
Ch. megacephala	Adult females	*S6k*	4	0.07558	0.01808	0.04683
Ch. albiceps	Larvae	*S6k*	1	0.02339	0.00614	0.01476
Ch. albiceps	Larvae	*S6k*	2	0.02296	0.00566	0.01431
Ch. albiceps	Larvae	*S6k*	3	0.01822	0.00618	0.01220
Ch. albiceps	Larvae	*S6k*	4	0.02113	0.04656	0.03385
Co. hominivorax	Larvae	*S6k*	1	0.02849	0.05826	0.04337
Co. hominivorax	Larvae	*S6k*	2	0.02737	0.04947	0.03842

Co. hominivorax	Larvae	*S6k*	3	0.02297	0.00923	0.01610
Co. hominivorax	Larvae	*S6k*	4	0.02281	0.00910	0.01595
Co. macellaria	Larvae	*S6k*	1	0.02665	0.03065	0.02865
Co. macellaria	Larvae	*S6k*	2	0.00903	0.03528	0.02215
Co. macellaria	Larvae	*S6k*	3	0.01745	0.00503	0.01124
Co. macellaria	Larvae	*S6k*	4	0.01393	0.00470	0.00932
Ch. megacephala	Larvae	*S6k*	1	0.02482	0.00587	0.01534
Ch. megacephala	Larvae	*S6k*	2	0.02113	0.00724	0.01419
Ch. megacephala	Larvae	*S6k*	3	0.02111	0.00484	0.01298
Ch. megacephala	Larvae	*S6k*	4	0.02883	0.00751	0.01817
Ch. albiceps	Adult males	*S6k*	1	0.02775	0.01036	0.01906
Ch. albiceps	Adult males	*S6k*	2	0.04823	0.02014	0.03418
Ch. albiceps	Adult males	*S6k*	3	0.03395	0.00770	0.02083
Ch. albiceps	Adult males	*S6k*	4	0.02776	0.00879	0.01827
Co. hominivorax	Adult males	*S6k*	1	0.07642	0.02825	0.05234
Co. hominivorax	Adult males	*S6k*	2	0.07848	0.03746	0.05797
Co. hominivorax	Adult males	*S6k*	3	0.07360	0.02349	0.04855
Co. hominivorax	Adult males	*S6k*	4	0.05779	0.02947	0.04363
Co. macellaria	Adult males	*S6k*	1	0.07870	0.04022	0.05946
Co. macellaria	Adult males	*S6k*	2	0.06933	0.02587	0.04760
Co. macellaria	Adult males	*S6k*	3	0.07667	0.02719	0.05193
Co. macellaria	Adult males	*S6k*	4	0.03321	0.01793	0.02557
Ch. megacephala	Adult males	*S6k*	1	0.05770	0.02192	0.03981
Ch. megacephala	Adult males	*S6k*	2	0.06598	0.02369	0.04484
Ch. megacephala	Adult males	*S6k*	3	0.05656	0.01659	0.03658
Ch. megacephala	Adult males	*S6k*	4	0.05335	0.01656	0.03496
Ch. albiceps	Adult females	*Sm*	1	0.01578	0.00989	0.01284
Ch. albiceps	Adult females	*Sm*	2	0.01975	0.01335	0.01655
Ch. albiceps	Adult females	*Sm*	3	0.04147	0.01009	0.02578
Ch. albiceps	Adult females	*Sm*	4	0.03752	0.01019	0.02385
Co. hominivorax	Adult females	*Sm*	1	0.01341	0.01028	0.01184
Co. hominivorax	Adult females	*Sm*	2	0.02234	0.01268	0.01751
Co. hominivorax	Adult females	*Sm*	3	0.05291	0.02308	0.03799
Co. hominivorax	Adult females	*Sm*	4	0.04884	0.02470	0.03677
Co. macellaria	Adult females	*Sm*	1	0.03991	0.01568	0.02779
Co. macellaria	Adult females	*Sm*	2	0.04402	0.01936	0.03169
Co. macellaria	Adult females	*Sm*	3	0.09019	0.02162	0.05591
Co. macellaria	Adult females	*Sm*	4	0.08445	0.02585	0.05515
Ch. megacephala	Adult females	*Sm*	1	0.01363	0.00679	0.01021
Ch. megacephala	Adult females	*Sm*	2	0.01346	0.00625	0.00986
Ch. megacephala	Adult females	*Sm*	3	0.03533	0.00988	0.02260
Ch. megacephala	Adult females	*Sm*	4	0.03638	0.01155	0.02397
Ch. albiceps	Larvae	*Sm*	1	0.00030	0.00007	0.00018
Ch. albiceps	Larvae	*Sm*	2	0.00032	0.00007	0.00019
Ch. albiceps	Larvae	*Sm*	3	0.00033	0.00011	0.00022
Ch. albiceps	Larvae	*Sm*	4	0.00039	0.00015	0.00027
Co. hominivorax	Larvae	*Sm*	1	0.00058	0.00119	0.00089
Co. hominivorax	Larvae	*Sm*	2	0.00077	0.00131	0.00104
Co. hominivorax	Larvae	*Sm*	3	0.00054	0.00017	0.00035
Co. hominivorax	Larvae	*Sm*	4	0.00046	0.00019	0.00033
Co. macellaria	Larvae	*Sm*	1	0.00503	0.00665	0.00584
Co. macellaria	Larvae	*Sm*	2	0.00315	0.00647	0.00481

Co. macellaria	Larvae	*Sm*	3	0.00085	0.00022	0.00053
Co. macellaria	Larvae	*Sm*	4	0.00105	0.00027	0.00066
Ch. megacephala	Larvae	*Sm*	1	0.00048	0.00009	0.00029
Ch. megacephala	Larvae	*Sm*	2	0.00025	0.00007	0.00016
Ch. megacephala	Larvae	*Sm*	3	0.00054	0.00013	0.00034
Ch. megacephala	Larvae	*Sm*	4	0.00058	0.00012	0.00035
Ch. albiceps	Adult males	*Sm*	1	0.02348	0.00985	0.01666
Ch. albiceps	Adult males	*Sm*	2	0.03150	0.01285	0.02217
Ch. albiceps	Adult males	*Sm*	3	0.05116	0.01105	0.03110
Ch. albiceps	Adult males	*Sm*	4	0.04893	0.00889	0.02891
Co. hominivorax	Adult males	*Sm*	1	0.05399	0.02228	0.03813
Co. hominivorax	Adult males	*Sm*	2	0.03411	0.01717	0.02564
Co. hominivorax	Adult males	*Sm*	3	0.07006	0.02276	0.04641
Co. hominivorax	Adult males	*Sm*	4	0.05171	0.02788	0.03979
Co. macellaria	Adult males	*Sm*	1	0.06343	0.02580	0.04462
Co. macellaria	Adult males	*Sm*	2	0.05433	0.02342	0.03887
Co. macellaria	Adult males	*Sm*	3	0.10066	0.02731	0.06399
Co. macellaria	Adult males	*Sm*	4	0.12334	0.03023	0.07679
Ch. megacephala	Adult males	*Sm*	1	0.02138	0.00668	0.01403
Ch. megacephala	Adult males	*Sm*	2	0.02892	0.00900	0.01896
Ch. megacephala	Adult males	*Sm*	3	0.04136	0.01255	0.02695
Ch. megacephala	Adult males	*Sm*	4	0.04182	0.01274	0.02728

MNE, Mean Normalized Expression
