# Supplementary material for: Evolution of genes involved in feeding preference and metabolic processes in Calliphoridae (Diptera: Calyptratae)
Source: PeerJ. 2016 Oct 27;4:e2598. doi: 10.7717/peerj.2598 (PMC5088637; doi:10.7717/peerj.2598)
Supplement: Table S7 [file peerj-04-2598-s007.pdf]

Table S7. Two-way ANOVA comparisons of gene expression levels.

Gene	Life stage	Factor	df	MS	SS	F	p-value
Cyp6g1	Adult females	Habit	1	0.00038364	0.00038364	5.822	0.03
		Species	2	0.00010122	0.00005061	0.768	0.49
	Larvae	Habit	1	0.02186200	0.02186150	4.653	0.05
		Species	2	0.00369900	0.00184950	0.394	0.68
	Adult males	Habit	1	0.00018397	0.00018397	4.620	0.05
		Species	2	0.00000945	0.00000473	0.119	0.89
for	Adult females	Habit	1	0.00036990	0.00036985	2.023	0.18
		Species	2	0.00617030	0.00308514	16.875	0.0003*
	Larvae	Habit	1	0.00028310	0.00028310	3.256	0.10
		Species	2	0.00070889	0.00035444	4.076	0.04
	Adult males	Habit	1	0.00026170	0.00026171	0.972	0.34
		Species	2	0.00266390	0.00133195	4.945	0.03
Gdh	Adult females	Habit	1	0.00002000	0.00001970	0.004	0.95
		Species	2	0.00256700	0.00128330	0.229	0.80
	Larvae	Habit	1	0.00017600	0.00017618	0.063	0.81
		Species	2	0.00602100	0.00301037	1.076	0.37
	Adult males	Habit	1	0.00071665	0.00071665	3.517	0.09
		Species	2	0.00092320	0.00046160	2.265	0.15
Jon65aiv	Adult females	Habit	1	0.09506500	0.09506500	11.596	0.009*
		Species	2	0.05473800	0.02736900	3.339	0.07
	Larvae	Habit	1	0.36380000	0.36376000	1.045	0.38
		Species	2	0.19080000	0.09538000	0.274	0.47
	Adult males	Habit	1	0.03193200	0.03193200	9.637	0.03
		Species	2	0.06156300	0.03078200	9.290	0.006*
Mvl	Adult females	Habit	1	0.09734600	0.09734600	9.719	0.00003*
		Species	1	0.06873600	0.03436800	3.431	0.60
	Larvae	Habit	1	0.00037269	0.00037269	48.307	0.00002*
		Species	1	0.00000217	0.00000108	0.140	0.87

	Adult males	Habit	1	0.00031396	0.00031396	20.563	0.0007*
		Species	1	0.00001764	0.00000882	0.578	0.58
PGRP-SC2	Adult females	Habit	1	0.00411180	0.00411180	5.194	0.04
		Species	2	0.01784750	0.00892370	11.272	0.002*
	Larvae	Habit	1	0.94600000	0.94602000	1.400	0.26
		Species	2	4.51220000	2.25612000	3.339	0.07
	Adult males	Habit	1	0.00296560	0.00296560	4.985	0.05
		Species	2	0.00662020	0.00331010	5.564	0.02
S6k	Adult females	Habit	1	0.00166460	0.00166463	5.822	0.03
		Species	2	0.00030900	0.00015452	0.540	0.60
	Larvae	Habit	1	0.00037610	0.00037610	3.749	0.08
		Species	2	0.00002806	0.00001403	0.140	0.87
	Adult males	Habit	1	0.00063351	0.00063351	7.831	0.02
		Species	2	0.00111552	0.00055776	6.894	0.01*
sm	Adult females	Habit	1	0.00000031	0.00000031	0.003	0.96
		Species	2	0.00161037	0.00080518	6.471	0.01*
	Larvae	Habit	1	0.00000075	0.00000075	0.387	0.55
		Species	2	0.00001959	0.00000980	5.037	0.03
	Adult males	Habit	1	0.00003264	0.00003264	0.280	0.61
		Species	2	0.00288753	0.00144377	12.381	0.001*

df, degrees of freedom; MS, mean squares; SS, sum of squares
*Significant expression differences: $p < 0.01$.
