# Supplementary material for: Evolution of genes involved in feeding preference and metabolic processes in Calliphoridae (Diptera: Calyptratae)
Source: PeerJ. 2016 Oct 27;4:e2598. doi: 10.7717/peerj.2598 (PMC5088637; doi:10.7717/peerj.2598)
Supplement: Table S8 [file peerj-04-2598-s008.pdf]

Table S8. Differences in gene expression between species pairs (Tukey HSD)

Gene	Life stage	Species	p-value
for	Adult females	*Co. hominivorax - Ch. albiceps*	0.09
		Co. macellaria - Ch. albiceps	0.22
		Ch. megacephala - Ch. albiceps	0.0005*
		Co. macellaria - Ch.hominivorax	0.95
		Ch. megacephala - Co. hominivorax	0.04
		Co. hominivorax - Co. macellaria	0.01*
Jon65aiv	Adult males	*Co. hominivorax - Ch. albiceps*	0.22
		Co. macellaria - Ch. albiceps	0.01*
		Co. hominivorax - Ch. albiceps	0.2
		Co. macellaria - Co. hominivorax	0.25
		Co. hominivorax - Co. hominivorax	1
		Co. hominivorax - Co. macellaria	0.29
PGRP-SC2	Adult females	*Co. hominivorax - Ch. albiceps*	0.25
		Co. macellaria - Ch. albiceps	0.58
		Co. hominivorax - Ch. albiceps	0.003*
		Co. macellaria - Co. hominivorax	0.91
		Co. hominivorax - Co. hominivorax	0.1
		Co. hominivorax - Co. macellaria	0.03
S6k	Adult males	*Co. hominivorax - Ch. albiceps*	0.23
		Co. macellaria - Ch. albiceps	0.02
		Co. hominivorax - Ch. albiceps	0.11
		Co. macellaria - Co. hominivorax	0.42
		Co. hominivorax - Co. hominivorax	0.97
		Co. hominivorax - Co. macellaria	0.69
sm	Adult females	*Co. hominivorax - Ch. albiceps*	0.84
		Co. macellaria - Ch. albiceps	0.06
		Co. hominivorax - Ch. albiceps	0.98
		Co. macellaria - Co. hominivorax	0.22
		Co. hominivorax - Co. hominivorax	0.62
		Co. hominivorax - Co. macellaria	0.03
	Adult males	*Co. hominivorax - Ch. albiceps*	0.61
		Co. macellaria - Ch. albiceps	0.01*
		Co. hominivorax - Ch. albiceps	0.98
		Co. macellaria - Co. hominivorax	0.06
		Co. hominivorax - Co. hominivorax	0.4
		Co. hominivorax - Co. macellaria	0.004*

*Significant expression levels differences: $p < 0.01$
