# Supplementary material for: Evolution of genes involved in feeding preference and metabolic processes in Calliphoridae (Diptera: Calyptratae)
Source: PeerJ. 2016 Oct 27;4:e2598. doi: 10.7717/peerj.2598 (PMC5088637; doi:10.7717/peerj.2598)
Supplement: Table S9 [file peerj-04-2598-s009.pdf]

Table S9. Tajima`s Relative Rate Test.

Gene	Outgroup	χ^2	p-value
for	*Ch. albiceps*	0.96	0.327
	Ch. megacephala	5.45	0.02
Gdh	*Ch. albiceps*	0.13	0.724
	Ch. megacephala	2.45	0.117
Mvl	*Ch. albiceps*	10.31	0.001
	Ch. megacephala	10.31	0.001
Jon65aiv	*Ch. albiceps*	0.04	0.835
	Ch. megacephala	0.04	0.835
s6K	*Ch. albiceps*	0	1
	Ch. megacephala	0.69	0.405

*Ingroup: *Co. hominivorax* e *Co. macellaria* in all comparisons
