# Supplementary material for: Evolution of genes involved in feeding preference and metabolic processes in Calliphoridae (Diptera: Calyptratae)
Source: PeerJ. 2016 Oct 27;4:e2598. doi: 10.7717/peerj.2598 (PMC5088637; doi:10.7717/peerj.2598)
Supplement: Table S10 [file peerj-04-2598-s010.pdf]

Table S10. d_n/d_s analysis using codeml.

Model	ω	Likelihood	X-squared	p-value
Neutral	1	-3944.34	------	------
Model1	0.02689	-3347.11	48.9181	2.67E-12
Model2	*Co. hominivorax*: 0.111<br>Others: 0.023	-3340.86	49.9896	1.55E-12
Model3	Obligate parasites: 0.027<br>Others: 0.027	-3347.1	48.9182	2.67E-12
Model4	Blood related: 0.027<br>Others: 0.027	-3347.1	48.9184	2.67E-12
